# Parasitism of Corn Earworm, *Helicoverpa zea* (Boddie) (Lepidoptera: Noctuidae), by Tachinid Flies in Cultivated Hemp

**DOI:** 10.3390/insects13060519

**Published:** 2022-06-03

**Authors:** Armando Falcon-Brindis, John O. Stireman, Zenaida J. Viloria, Raul T. Villanueva

**Affiliations:** 1 Research and Education Center, University of Kentucky, 348 University Drive, Princeton, KY 42445, USA; armando.falcon.brindis@uky.edu (A.F.-B.); zenaida.viloria@uky.edu (Z.J.V.); 2 Biological Sciences Bldg. 020, Wright University, 3640 Colonel Glenn Hwy., Dayton, OH 45435, USA; john.stireman@wright.edu

**Keywords:** biological control, Tachinidae, bristle fly, *Cannabis sativa*, superparasitism, *Winthemia*, *Lespesia*

## Abstract

**Simple Summary:**

Hemp has become a rapidly growing industry in the United States in recent years. However, due to many decades of prohibition, there has been relatively little research on insect pests and their interactions with natural enemies in hemp production systems. Here, we provide the first quantitative assessment of corn earworm (CEW) *Helicoverpa zea* parasitism in a hemp system. Corn earworm larvae exhibited high parasitism rates by tachinid flies resulting in elevated mortality. Host mortality increased with the number of tachinid eggs per larva even though typically only one parasitoid successfully developed per host. Larger CEW larvae were more likely to survive parasitism, but frequently, neither parasitoid nor host larvae successfully developed. Our results suggest that tachinid flies hold promise as biological control agents for populations of this important pest attacking hemp.

**Abstract:**

In a survey on hemp grown in western Kentucky we found an average of 27.8 CEW larvae per plant. We recorded 45% parasitism of CEW in these fields by two species of tachinid flies, *Winthemia rufopicta* and *Lespesia aletiae*. Most parasitized larvae were third to sixth instars at the time of collection. We found up to 22 tachinid eggs per host larva, 89% of which typically bore between 1 and 5 eggs on the thorax. 45.9% of CEW bearing eggs died. The number of tachinid eggs per host was unrelated to host body mass, but both the number of tachinid eggs and caterpillar body mass influenced CEW survival. Larger CEW often survived parasitism and the number of fly eggs was negatively related to survival rate. The emergence of adult flies was positively correlated with the number of eggs, but no influence of the host size was found. High mortality of CEW larvae and the parasitoids developing within them in this system suggests that secondary chemicals (or poor nutrition) of the hemp diet may be negatively affecting host and parasitoid development and influencing their interactions.

## 1. Introduction

The cultivation of hemp (*Cannabis sativa* L.) has had a long and complex history in the U.S. since it was brought to the Americas by early colonists [1]. Hemp was widely grown as a fiber crop in the 18th, 19th and early 20th centuries, and domestic production peaked in 1943 in an effort to support the United States’ involvement in World War II. However, hemp production dramatically declined in the middle of the century due to drug enforcement laws and competition with other fibers [2]. After the resurgence of the hemp industry in the United States, allowed by the Agricultural Act of 2014, the production of *Cannabis sativa* with <0.3% of delta-9-tetrahydrocannabinol started to rise. By 2018, about 78,000 acres across 23 states had been licensed to grow hemp, and the following year, the number of licensed acres exponentially increased to 500,000 across 45 states [3]. To target a growing market for products related to human nutrition, medicine, cosmetics, textiles, paper, and fuel, the production of hemp has diversified in at least 25,000 commercial products [4]. This has positioned hemp as a promising crop to spur domestic economic growth [5].

Despite the potential of agricultural hemp, many decades of prohibition have stifled research on the management of insect pests and their impacts on cultivated hemp [6]. More than 270 species have been documented as feeding on *C. sativa* around the world, including defoliators, chewing, and sucking insects and mites, stem and stalk borers, and root feeders [7,8,9]. Among this diversity of potential pests, lepidopterous stem borers (e.g., *Ostrinia nubialis*) and budworms (e.g., *Heliothis* spp., *Helicoverpa* spp.) have been singled out as the most recurrent and severe problems in hemp field crops across the United States [10]. In particular, the corn earworm (CEW) *Helicoverpa zea* (Boddie) (Lepidoptera: Noctuidae) is a prevalent pest on hemp that can reach outbreak densities [11]. This species is a rising concern among hemp growers who have reported infestations in Colorado, Illinois, Indiana, Kentucky, Nevada, North Carolina, Tennessee, Vermont, Virginia, and Wisconsin [2,12]. Because CEW feeds on flower buds, the most valuable part of cultivars grown to produce cannabidiol, it is considered a key pest of hemp.

Controlling hemp pests in the United States is regulated and restricted to the use of biopesticides, such as bacteria, viruses, and plant extracts [13]. The release of natural enemies is a common practice for indoor hemp production, but research on the classical biological control of CEW in hemp is still limited. Although at least 17 families of arthropods have been listed in the United States as natural enemies of hemp pests [11], the impact of most of them on hemp cultivation remains unclear. Subsequently, the role of natural enemies in controlling pests like CEW in these systems is largely unexplored [14], and no studies have thus far examined the bionomics of tachinid fly parasitoids (i.e., “bristle flies”) in these systems.

Previous research examining the interactions between tachinid parasitoids and CEW has demonstrated that they can be important enemies of CEW in other agricultural systems [15,16,17,18]; however, no previous research has examined these interactions in cultivated hemp. In the present work, we aimed to document the tachinid community associated with CEW in hemp, assess parasitism frequencies, and explore the bionomics of CEW-tachinid interactions in hemp agroecosystems. To tackle these goals, we addressed specific questions: (1) What parasitoids are present and what levels of parasitism do CEW experience in hemp agricultural systems? (2) How much CEW mortality do tachinid parasitoids cause and is this related to host size or the number and placement of parasitoid eggs? (3) How does host size and the number of eggs influence the adult emergence of tachinid parasitoids?

We predicted that greater CEW body mass would increase the likelihood of survival of parasitized larvae due to more effective immune defenses (e.g., encapsulation of larval parasitoids) and a greater probability that CEW larvae could complete development before parasitoid larvae could kill them [19,20]. Conversely, smaller, earlier instar CEW larvae with shorter inter-molt periods could have an advantage against parasitoids because they may be more likely to molt off externally deposited tachinid eggs before they hatch [17]. We further predicted that greater numbers of fly eggs per larvae would decrease the chances of survival of both hosts and parasitoids due to the overwhelming of host defenses and larval competition for resources, respectively [16,21].

## 2. Materials and Methods

### 2.1. Field Work

**Figure 1:**
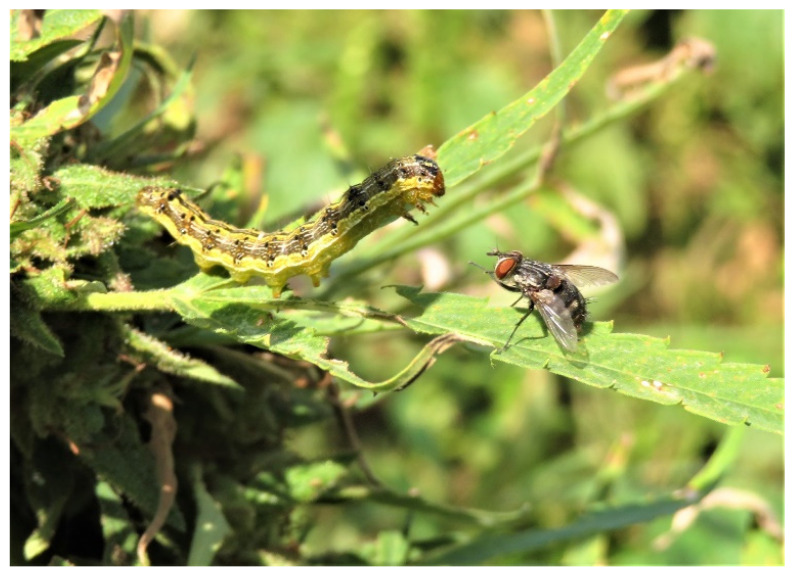

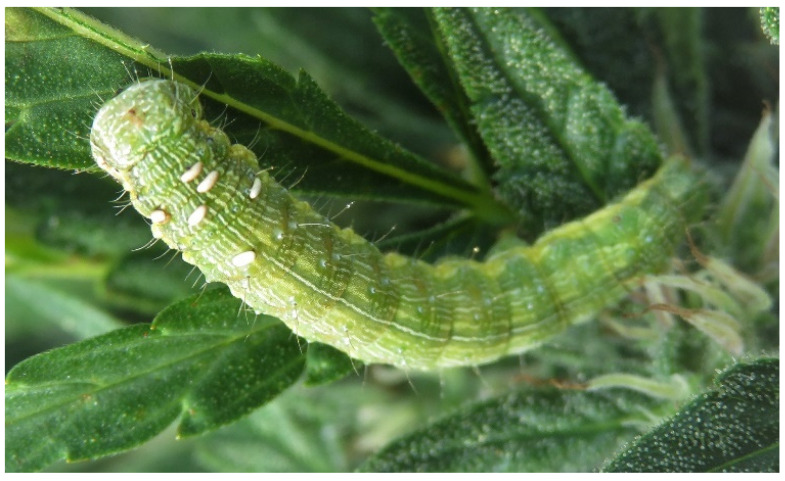
*Winthemia rufopicta* preparing to lay eggs on a CEW larva (**top**). Caterpillar of *H. zea* with attached eggs of *W. rufopicta* on the thorax (**bottom**).

Field sampling took place in three hemp farms located in Caldwell (2 farms) and Calloway counties (1 farm) of Western Kentucky, United States. Farms varied in size and management and were surrounded by different vegetation cover. In late August 2021, we observed large numbers of CEW larvae feeding on flower buds in these hemp farms. Along with the CEW larvae, many adult tachinid flies were observed in situ flying, walking on hemp plants, and ovipositing on CEW caterpillars (Figure 1). From September to October 2021, CEW caterpillars were sampled from unsprayed hemp plants in each commercial hemp farm. In total, we collected 276 caterpillars of which 200 were apparently parasitized (i.e., they bore conspicuous tachinid eggs attached to the body, Figure 1) and 76 were apparently unparasitized (no eggs) or healthy. The collected caterpillars were taken to the laboratory of Entomology at the University of Kentucky, Research & Education Center, Princeton, KY for further inspection and rearing.

To estimate the proportion of healthy and parasitized larvae in the field, we randomly selected individual plants (*N* = 390) and quantified the number of caterpillars per plant and the proportion bearing visible tachinid eggs. This assessment was conducted at Caldwell Farm 1 during September and October. In addition to CEW, several yellow-striped armyworm caterpillars, *Spodoptera ornithogalli* (Guenée), were observed bearing tachinid eggs, but they were not examined in this study.

### 2.2. Laboratory Measurements

In the laboratory, the caterpillars were carefully weighed and the number of attached tachinid eggs was recorded. Since the tachinid eggs appeared to be distributed nonrandomly across the caterpillar bodies, we categorized the location of tachinid eggs on CEW larvae as either the thorax, middle abdomen, or posterior abdomen (no eggs were observed on head capsules).

Corn earworm larvae were individually placed in plastic cups (59.1 mL) that were covered with a perforated lid to avoid water condensation. Monitoring was conducted every 1–2 days until the death of larva, or the emergence of CEW moths or tachinid flies. Caterpillars were fed ad libitum with buds and leaves from healthy hemp plants. All CEW larvae were kept in a growing chamber set at 25 °C, 20%RH, photoperiod of 12:12 (L:D). Adult tachinids were identified using the keys of Guimarães [22] and Sabrosky [23,24], along with a comparison with specimens in the JOS collection at Wright State University (Dayton, OH, USA). Distinct differences were observed in the spiracular morphology of the reared tachinid species [25], allowing the classification of un-enclosed puparia as well as adult flies. Voucher specimens were deposited at the JOS collection and the insect collection of the University of Kentucky.

### 2.3. Data Analysis

We used two independent variables as predictors explaining the mortality of CEW larvae: the body mass of larva and the number of apparent tachinid eggs it bore. We used body mass (g) as a proxy of the larval instar. The stadia of CEW caterpillars were categorized from L1 to L6 according to Hardwick [26], where larval lengths are estimated to be on average 1.5, 3.4, 7.0, 11.4, 17.9, and 24.8 mm for each instar, respectively. To associate Hardwick’s category with weight, we measured and weighed the length and body mass of 50 caterpillars when collected. We did not measure body length daily to avoid excessive manipulation of the larva and detachment of the tachinid eggs. The number of fly eggs on the host was quantified to evaluate the relationship between egg number and caterpillar survival, as well as egg number and the number of parasitoids emerging from a host [27].

### 2.4. Mortality of CEW and Tachinid Emergence

The percentage of CEW mortality was estimated in both healthy and parasitized caterpillars using Henderson-Tilton’s formula of corrected mortality [28]. We used binomial Generalized Linear Models (GLM) to estimate the probability of survival of CEWs and the success of tachinid flies in relation to host body mass and number of tachinid eggs. The odds ratio (OR) of each predictor was also calculated where OR values >1 indicated a greater probability of survival and OR values < 1 indicated a lower probability of survival. The overall effect of each variable was evaluated using Wald’s test. ANOVA was used to compare models and assess if individual explanatory variables explained significant variance in survival. We used a regression analysis to test the relationship between the caterpillar mass (g) and the number of fly eggs and between mean number of tachinid flies in response to the number of eggs per host. Model fitting and regressions were conducted in R v4.1.1 [29]. The package *MASS* was used to estimate and plot predicted mortality [30]. We conducted a survival analysis using the Kaplan-Meier function to test whether the intensity of parasitism (i.e., the number of tachinid eggs) affected the probability of the survival of CEW larvae over time [31]. In addition, we discretized the number of fly eggs per host larva to test whether there was a threshold in the survival in response to the level of parasitism (low: 1–5, moderate: 6–10, or high: >10), and to evaluate possible preferences of female flies for greater CEW size. This approach was also implemented by Danks [17] using different egg rank categories. Censored individuals corresponded to those who failed (caterpillars that neither pupated nor produced tachinid puparia) at any stage. The Kaplan–Meier nonparametric estimation was conducted in R using the survival package [32].

## 3. Results

### 3.1. Parasitism Frequency

In surveyed hemp fields, the mean ± SEM number of CEW per plant was 27.8 ± 3.7, ranging from 16 to 49. The overall parasitism rate in the field was 45%. An average of 42% of CEW larvae were parasitized per hemp plant. Of the parasitized caterpillars, 50% were parasitized by *Winthemia rufopicta*, 40% by *Lespesia aletiae,* and in 10% of the cases, both flies emerged from the same host. However, typically (86%), only a single fly larva emerged from a host, and we never observed more than two larvae emerging from a host. Most larvae of both fly species (90%) emerged from the larval stage of the host, and 10% emerged from moth pupae.

**Figure 2:**
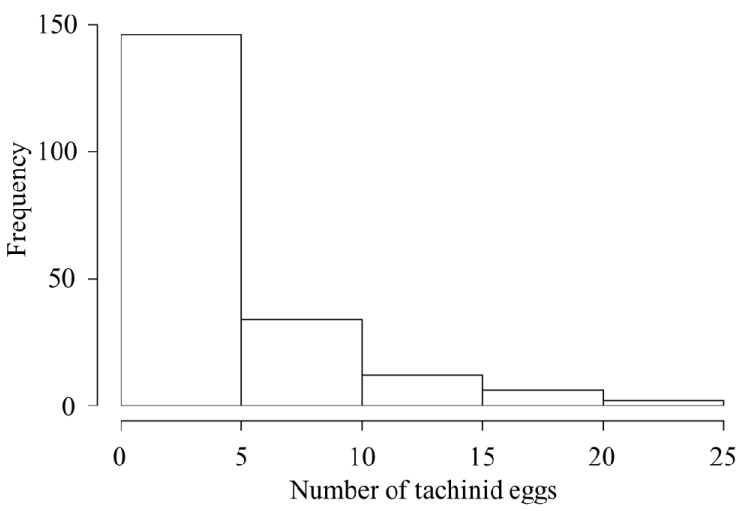
Histogram of visible tachinid eggs per host.

**Figure 3:**
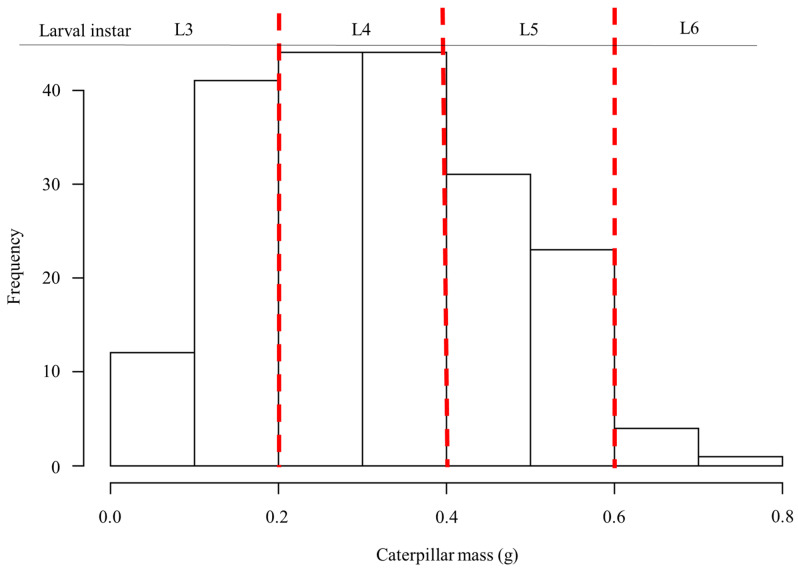
Distribution of collected CEW larvae according to their body mass and categorization of larval instar. Red dashed lines indicate approximate boundaries between instars.

The total number of tachinid eggs observed per parasitized CEW larva ranged from 1 to 22, most frequently (73% of the individuals) between 1 and 5 eggs per larva (Figure 2, Table 1). The mean body mass of healthy and parasitized caterpillars was not significantly different (t = −1.76, df = 45.6, *p* = 0.085). The mass of parasitized individuals averaged 0.32 g at the time of collection, ranging from 0.02 g to 0.71 g (Table 1), corresponding to L3–L6 (Figure 3). The most frequently attacked caterpillars were in the fourth instar (Figure 3).

**Figure 4:**
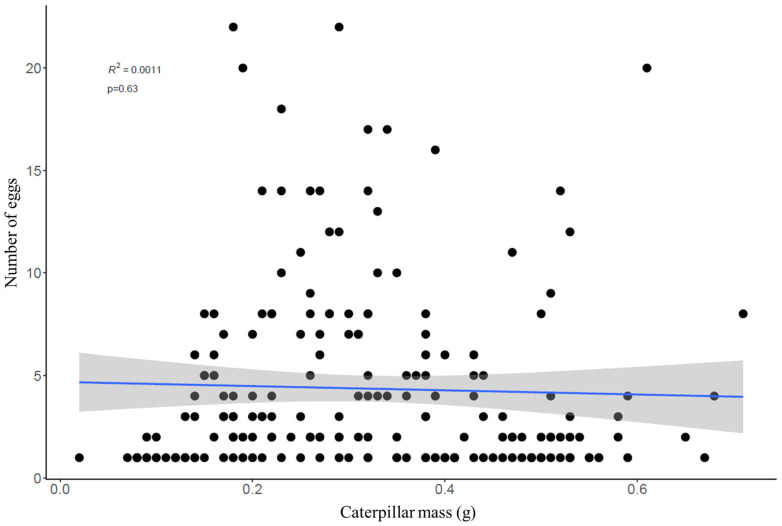
Scatterplot showing the relationship between the caterpillar weight and the number of tachinid eggs. The blue line indicates the linear model and its standard error (grey fill).

The number of tachinid eggs per host was unrelated to host mass (Figure 4, t = −0.47, df = 198, *p* = 0.63), but varied significantly across body regions (F = 154.5, df = 2, *p* < 0.001), with 89% of eggs located on the thorax region. There was no relationship between the body mass of CEW larvae and the number of eggs on each of the body regions (thorax, middle, posterior).

### 3.2. Mortality

**Figure 5:**
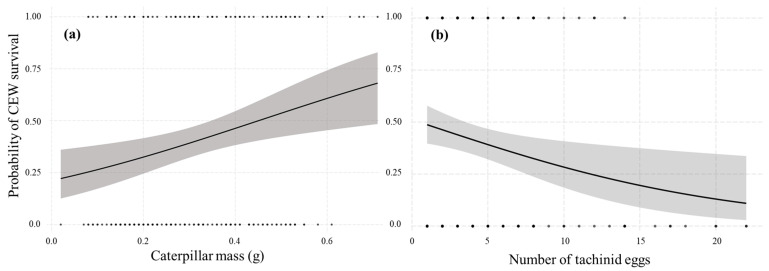
Predicted probability of CEW survival with respect to body mass (**a**) and number of tachinid eggs per host (**b**). Grey areas indicate the standard error.

**Figure 6:**
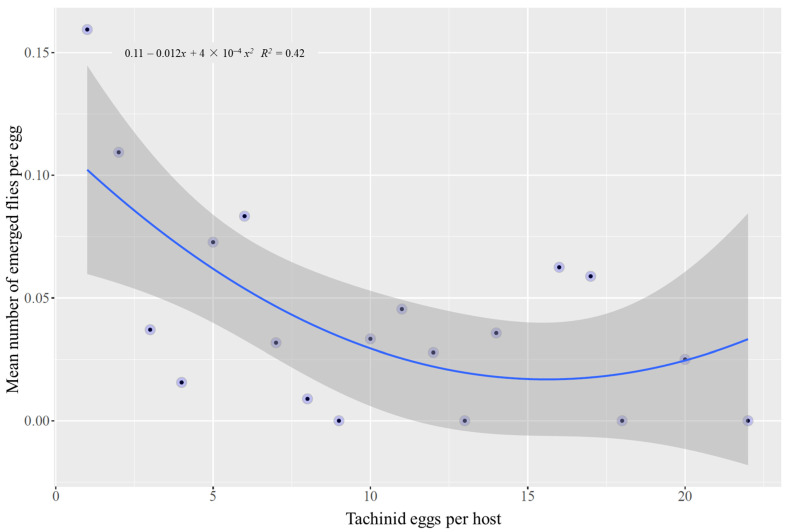
Relationship between the mean number of tachinid flies in response to the number of eggs per host.

Overall, 45.9% of CEW-bearing eggs died, while mortality of the unparasitized larva reached 19.1%. The percentage of parasitized larvae that produced parasitoid puparia ranged between 17% and 85% among collections, with the highest mortality of parasitized CEW in late October (Table 2). Mortality differed significantly between larvae with and without tachinid eggs (χ^2^ = 25.9, df = 1, *p* < 0.001). According to our logistic model (Table 3), both the number of tachinid eggs and caterpillar body mass influenced CEW survival (Wald test, χ^2^ = 14.9, df = 2, *p* < 0.001). Caterpillar survival increased with caterpillar mass (Figure 5a). Larger CEW (0.35 ± 0.15 g) often survived parasitism, whereas smaller caterpillars (0.28 ± 0.16 g) were more likely to succumb to the developing parasitoids. The number of tachinid eggs was negatively related to the probability of CEW survival: the more fly eggs, the lower the probability of survival (Figure 5b). The success of parasitoids (emergence of adults) was positively correlated with the number of eggs, but no influence of the host size was found (Table 3). In contrast, the mean number of tachinids emerging per egg (per capita survival of parasitoids) decreased as the number of eggs on the host increased (Figure 6).

**Figure 7:**
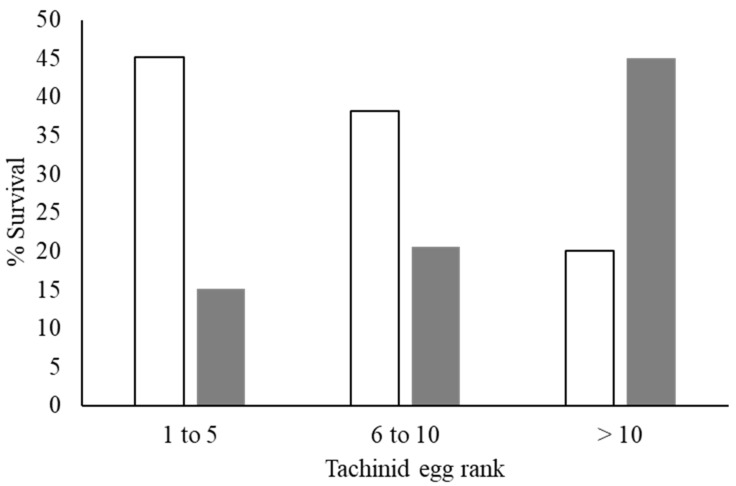
Responses in the survival of CEW (white bars) and adult flies (grey) across tachinid egg rank categories. Each egg category shows the percentage of successful CEW, and the proportion of adult flies emerging from the dead individuals.

**Figure 8:**
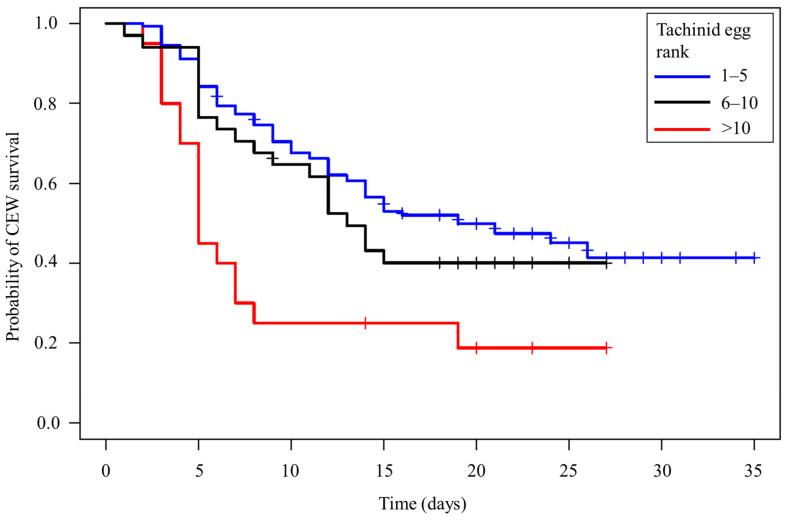
Kaplan–Meier curves of CEW larval survival relative to egg rank category. Perpendicular lines indicate censored individuals.

Host mortality and adult tachinid emergence varied significantly among parasitoid egg rank categories (χ^2^ = 28.6, df = 4, *p* < 0.001), with the lowest host survival and highest proportion of adult fly emergence when >10 eggs were present on the CEW larva (Figure 7). The median survival time of parasitized CEW larvae was 15 days. Survival time was associated with the number of parasitoid eggs. For caterpillars with 1–5, 6–10, and >10 parasitoid eggs, the median survival time was 19, 13, and 5 days, respectively (Figure 8). The divergence of the three survival curves was statistically significant (χ^2^ = 14.9, df = 2, *p* < 0.001).

## 4. Discussion

### 4.1. Parasitism

Here, we provide the first quantitative assessment of tachinid parasitism of CEW feeding on hemp, a growing industry in the United States. In the hemp fields surveyed, we found that 45% of CEW larvae were apparently parasitized by tachinids based on the presence of eggs of *W. rufopicta*. This likely represents an underestimate as larvae without obvious eggs may also host parasitoids, either of other taxa that do not leave behind conspicuous eggshells or due to the loss of these eggs through caterpillar molting. Interestingly however, no tachinids emerged from caterpillars that lacked parasitoid eggs, nor were any hymenopteran parasitoids reared from the collected larvae.

Both tachinid parasitoids reared in this study have been recorded previously from *H. zea* [16,17,25,33] and are known to attack a variety of noctuid caterpillars, as well as taxa in other lepidopteran families [34]. While *W. rufopicta* appears to primarily attack generalist noctuids like CEW, *L. aletiae* has been reared from a wide variety of caterpillars belonging to at least 15 families [34,35,36]. As indicated previously, *W. rufopicta* is oviparous, laying hard shelled, unembryonated eggs that must develop for several days before the larva hatches and burrows into the host, whereas *L. aletiae* deposits thin-shelled, membranous eggs that are fully developed (ovolarvipary) and hatch within minutes of deposition [37]. These latter eggs are typically not apparent on hosts even shortly after oviposition. Therefore, detecting, and quantifying attacks from *L. aletiae* can be challenging without conducting species-specific rearing experiments.

Interestingly, we found no evidence of such “cryptic parasitism.” That we only reared *L. aletiae* from CEW caterpillars that were also parasitized by *W. rufopicta* (i.e., that bore eggs) is a surprising pattern. Given the relatively low numbers of caterpillars lacking obvious tachinid eggs that were collected (*N* = 76), it is possible that this is simply due to chance, but with about 8.5% of (*W. rufopicta*) egg-bearing tachinids producing *L. aletiae*, this appears unlikely (*p* < 0.01). This could be explained by individual caterpillars apparent to *Winthemia* also being apparent to *Lespesia* due to their microhabitat, activity level, or other traits. It is also possible that prior parasitism by *Winthemia* weakens host defenses sufficiently to allow *Lespesia* to successfully develop, where they would otherwise succumb to encapsulation or other defenses. In either case, it appears that *Lespesia* could be the dominant competitor when both species are present in a host, as this species dominated the parasitism of caterpillars bearing *Winthemia* eggs, at least later in the season.

### 4.2. Parasitoid Egg Numbers and Distribution

**Figure 9:**
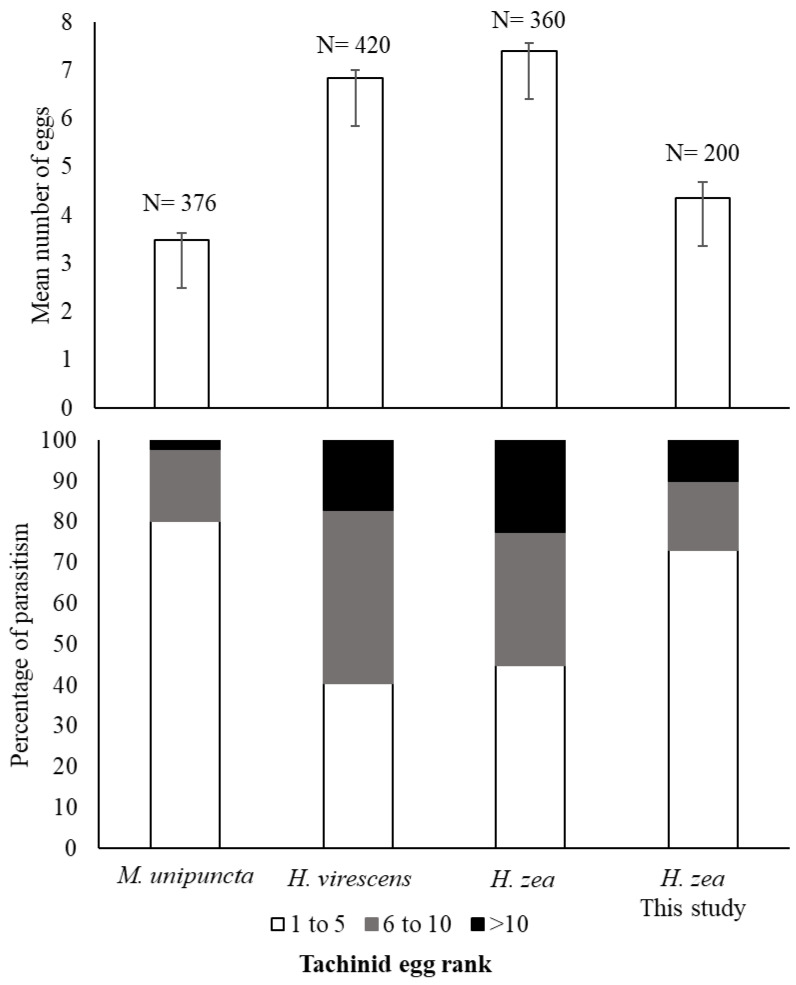
Parasitism of *W. rufopicta* across different host species. Mean number of eggs per host (upper chart) and percentage of parasitism (bottom). *N* = the number of caterpillars in each experiment. Whiskers indicate the SE of the mean. Data from *M. unipunctata* were obtained from Sherman [38], and those on *H. virescens* and *H. zea* were summarized from Danks [17].

The observed numbers of eggs per host that were laid by *W. rufopicta* in this study was comparable to previous studies [16,17,38] in other crop systems. For example, Sherman [38] found that parasitized caterpillars of *Mythimna unipuncta* Haworth (Noctuidae) in North Carolina bore between 1 and 15 attached eggs (mean ± SD 3.5 ± 2.6) of *W. rufopicta*. Danks [17] reported between 1 and 24 eggs of *W. rufopicta* on *H. virescens* and *H. zea* larvae attacking tobacco plants. Our results agree with these previous studies in that most larvae bear between 1 and 5 eggs of *W. rufopicta*, with estimates of the mean number of parasitoid eggs per individual between 3 and 7 (Figure 9).

We found a strong preference of *W. rufopicta* to lay eggs on the thorax of the host, which agrees with a previous study of parasitism of CEW by *W. rufopicta* under laboratory conditions [17]. Similar behavior has been documented in the exotic tachinid *Drino munda* (Wiedemann) parasitizing CEW [39] and *Exorista flaviceps* Macquart attacking *Pieris rapae* (L.) [40], among others. Oviposition stimulants may proximately explain this pattern, given that some tachinids that lay eggs directly on the host are known to rely on physical stimuli, including visual cues [41,42], and host attributes such as color, texture, and movements can influence the oviposition behavior [43,44]. Indeed, during the field sampling we observed female flies trying to oviposit on the thoracic area of CEW larvae, apparently following the movement of the host head (Videos S1 and S2). Ultimately, the strategy is likely adaptive, as eggs that are laid on the thorax cannot be bitten or removed by the host before they are able to develop and hatch [17,45]. Other species of caterpillars have been observed biting and destroying tachinid eggs with their mouthparts (e.g., [44]).

### 4.3. Host Mortality and Parasitoid Success

According to our first prediction, the positive relationship between the CEW body mass and survival may be attributed to more effective immune defenses (e.g., encapsulation of larval parasitoids) and a greater probability that large CEW larvae could complete development before the parasitoid larvae could kill them [19,20]. In contrast, host size did not explain the success of parasitoids (emergence of adult flies). This finding differs from a previous study on *Exorista thula* Wood parasitizing *Gynaephora groenlandica* (Wocke) and *G. rossii* (Curtis) [46], where the success of *E. thula* was positively correlated with the size of both host species.

Our second prediction, that greater egg numbers per host would lead to more intense competition among parasitoid larvae and thus lower parasitoid success, was not met. Instead, we observed an increased probability of fly emergence with greater numbers of fly eggs per host, as well as a positive relationship between the number of fly eggs and host mortality. However, the per capita (per egg) emergence rate of tachinids declined with increasing eggs, indicating a cost of superparasitism. Regardless of how many parasitoids a caterpillar bore, typically only one (occasionally two) larva emerged. Previous research has indicated that superparasitism can reduce the success of both *W. rufopicta* and *L. aletiae* [17,37]. In fact, the average puparia weight of *L. aletiae* has been shown to decrease with an increasing number of conspecific eggs [37]. These studies were conducted in different agricultural systems (i.e., tobacco, corn, soybean) and used artificial diets to feed the parasitized larvae. Our contrasting results may be due to interactions between the hemp diet, immune defenses of CEW, and parasitoid competition.

**Figure 10:**
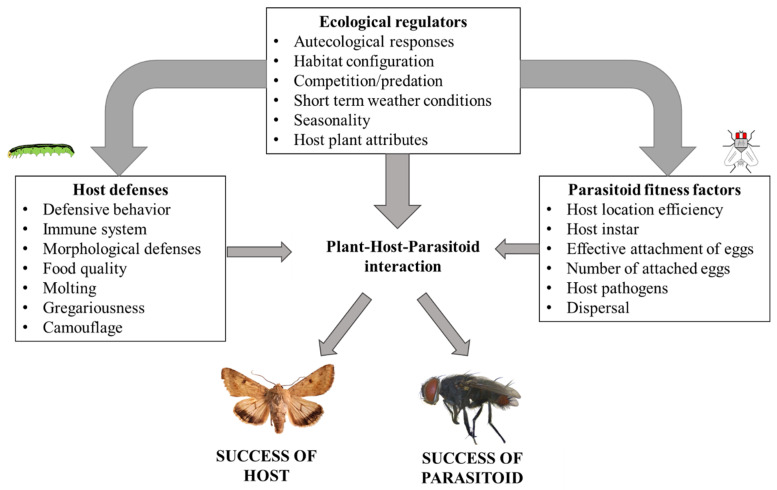
Simplified diagram of potential factors influencing the success of CEW and tachinid parasitoids. Arrows show the direction of regulation, where ecological factors, parasitoid fitness and host defenses influence the outcome of the tritrophic interaction. More complexity could be included since factors interact to determine fitness outcomes of plant-host-parasitoid interactions.

Caterpillar-tachinid interactions can be influenced by many different factors that are associated with each trophic level and the environment (Figure 10). Previous research examining tachinid-host interactions supports the idea of multidimensional drivers explaining the success of either the host or parasitoid [35,41,47,48,49]. In this work, given that the *L. aletiae* eggs were imperceptible on the studied CEW larvae, we were not able to quantify the mortality per tachinid species, thus adding complexity to the interpretation of host mortality and parasitoid success. Instead, we could only evaluate parasitoid success and mortality based on the number of eggs of *W. rufopicta.* In this sense, the mortality of CEW as evaluated here should be considered the absolute mortality due to tachinids. In other systems (e.g., either natural or artificial), where tachinids and parasitic wasps share the same hosts, the interactive effects of parasitoids on host mortality may be even more complex [50,51]. The absence of parasitic wasps attacking CEW in this hemp system is unexpected since several species of braconid, ichneumonid, and trichogrammatid wasps have been reported as parasitoids of CEW in the United States [52,53]. In fact, parasitic wasps can cause greater mortality of CEW than *W. rufopicta* in soybean systems [54]. However, hymenopteran parasitoids often attack early developmental stages of Lepidoptera and may have been missed by our focus on later instars [55]. Future research on the CEW mortality and parasitoid success should include a broader range of developmental stages and replication across different local and landscape conditions.

The hemp diet is likely a key factor modulating the mortality of parasitized CEW and tachinid success and may help to explain the relatively low parasitoid success rates in this system. Previous studies on the parasitism of *W. rufopicta* on *H. zea*, and *L. aletiae* on *Syntomeida epialis* (Walker) (Erebidae) and *Spodoptera frugiperda* (Smith) (Noctuidae) used artificial diets to feed both the caterpillars and flies [16,17,37], thereby altering the performance of both host and parasitoid by providing different conditions than those found in the field. Although artificial diets are convenient to conduct behavioral experiments and may provide important biological data, the use of host plants in the diet may be more realistic in terms of the plant-host-parasitoid interaction occurring in the field [56]. For example, secondary plant compounds may impair caterpillar physiological defenses [57] or compromise the fitness of immature parasitoids [58,59], as might be the case in hemp. It has been observed that the survival and sex ratio of *Helicoverpa armigera* (Hübner) varied significantly among different cultivated host plants [60], and the capacity of caterpillars to encapsulate foreign bodies may also vary according to the food source [20]. It may be insightful to evaluate the influence of the diet (hemp versus artificial) on the mortality of CEW and the success of tachinid flies.

## 5. Conclusions

In summary, we found that a large proportion (45%) of CEW larvae bore tachinid eggs in an outbreaking CEW population in hemp fields in Western Kentucky. Two species of generalist tachinids were reared in this system, *W. rufopicta* and *L. aletiae*, but no hymenopteran parasitoids were recovered. The number of eggs was unrelated to caterpillar size, and most were located on the thoracic region of CEW larvae. In the laboratory, CEW larvae with eggs experienced significantly greater mortality than those without; however, the apparent high parasitism frequency did not translate into large numbers of parasitoid adults, with only 9% of caterpillars bearing parasitoid eggs that produced adult flies. Overall host survival was positively related to body mass and negatively related to the number of tachinid eggs. Tachinid survival was positively related to the number of fly eggs on the host. Conversely, the mean number of adult flies emerging from the host decreased as the number of tachinid eggs increased. Typically, only one fly emerged from a host, indicating strong competition among larvae, but the positive association between egg number per host and the likelihood of producing an adult tachinid suggests a facilitative effect. The high mortality of CEW larvae and developing tachinid larvae in this system suggests that secondary chemicals (or poor nutrition) in the hemp diet may be negatively affecting host and parasitoid development and influencing their interaction. Still, the high parasitism rates that we observed, and the elevated mortality of the parasitized CEW suggest that these tachinid flies may exert significant biological control over this important pest in hemp.

## Figures and Tables

**Table 1:** Means, standard errors and ranges of CEW caterpillar body mass and tachinid egg numbers.

Variables	Mean	±SE	Range (Min–Max)
Body mass	0.32 g	0.01	0.02–0.71 g
Tachinid eggs/host	4.36	0.31	1–22
No. of tachinid eggs on thorax	4.03	0.29	0–20
No. of tachinid eggs on middle	0.33	0.06	0–5
No. of tachinid eggs on posterior	0.12	0.03	0–3

**Table 2:** Mortality of parasitized caterpillars over sampling events. *N* corresponds to the number of parasitized caterpillars collected at each date.

Date	*N*	Mortality (%)	Tachinid Species
14 September 2021	46	33	*W. rufopicta* (52%), *L. aletiae* (48%)
17 September 2021	11	82	*W. rufopicta*
20 September 2021	38	71	*W. rufopicta*
23 September 2021	20	75	*W. rufopicta*
27 September 2021	24	83	*W. rufopicta* (57%), *L. aletiae* (43%)
01 October 2021	6	17	*L. aletiae*
20 October 2021	55	85	*W. rufopicta* (14%), *L. aletiae* (86%)

**Table 3:** Summary of the logistic regression models explaining the survival of CEW and success of tachinid flies (emergence of adults). SE = standard error, OR = odds ratio. Significant at 0.01 (*), 0.001 (**), <0.001 (***).

Survival/Success	Variable	Estimate	SE	*p*-Value	OR
CEW	Intercept	−0.89	0.392	0.023 *	0.41
	CEW body mass	2.91	1.013	0.003 **	18.52
	Number of tachinid eggs	−0.09	0.038	0.011 *	0.91
Adult flies	Intercept	−3.21	0.748	<0.001 ***	0.04
	CEW body mass	−0.52	1.985	0.793	0.59
	Number of tachinid eggs	0.16	0.043	<0.001 ***	1.18

## Data Availability

The data presented in this study are available on request from the corresponding author.

## Supplementary Materials

The following supporting information can be downloaded at: https://www.mdpi.com/article/10.3390/insects13060519/s1, Video S1: *L. aletiae*; Video S2: *W. rufopicta*.
